# Improvement of sensory neuron growth and survival via negatively regulating PTEN by miR-21-5p-contained small extracellular vesicles from skin precursor-derived Schwann cells

**DOI:** 10.1186/s13287-020-02125-4

**Published:** 2021-01-25

**Authors:** Meng Cong, Mi Shen, Xia Wu, Yan Li, Liting Wang, Qianru He, Haiyan Shi, Fei Ding

**Affiliations:** 1 School of Biology and Basic Medical Sciences, Soochow University, Suzhou, 215123 China; 2 Key Laboratory of Neuroregeneration of Jiangsu and Ministry of Education and Co-innovation Center of Neuroregeneration, Nantong University, 19 Qixiu Road, Nantong, 226001 China; 3 Department of Pathophysiology, School of Medicine, Nantong University, 19 Qixiu Road, Nantong, 226001 China; 4 Jiangsu Clinical Medicine Center of Tissue Engineering and Nerve Injury Repair, Affiliated Hospital of Nantong University, 20 Xisi Road, Nantong, 226001 China

**Keywords:** Skin precursors, Schwann cells, Extracellular vesicles, Sensory neurons, PI3K/Akt, MicroRNAs, PTEN, Growth, Survival

## Abstract

**Background:**

Patients with peripheral nerve injury (PNI) often suffer from hypoxic ischemic impairments, in particular when combined with vascular damage, causing neuronal dysfunction and death. Increasing attention has been paid on skin precursor-derived Schwann cells (SKP-SCs), and previous study has shown that SKP-SCs could promote sensory recovery after cell therapy for PNI, resembling the effect of naive SCs, and SKP-SC-derived extracellular vesicles (SKP-SC-EVs) are putatively supposed to be promising therapeutic agents for neural regeneration.

**Methods:**

SKPs were induced to differentiate towards SCs with cocktail factors (N2, neuregulin-1β, and forskolin) in vitro. SKP-SC-EVs were isolated by exoEasy Maxi Kit and characterized by morphology and phenotypic markers of EVs. Rat sensory neurons from dorsal root ganglions (DRGs) were primarily cultured in regular condition or exposed to oxygen-glucose-deprivation (OGD) condition. SKP-SC-EVs were applied to DRGs or sensory neurons, with LY294002 (a PI3K inhibitor) added; the effect on neurite outgrowth and cell survival was observed. Moreover, microRNA (miR) candidate contained in SKP-SC-EVs was screened out, and miR-mimics were transfected into DRG neurons; meanwhile, the negative regulation of PTEN/PI3K/Akt axis and downstream signaling molecules were determined.

**Results:**

It was shown that SKP-SC-EVs could improve the neurite outgrowth of DRGs and sensory neurons. Furthermore, SKP-SC-EVs enhanced the survival of sensory neurons after OGD exposure by alleviating neuronal apoptosis and strengthening cell viability, and the expression of GAP43 (a neuron functional protein) in neurons was upregulated. Moreover, the neuro-reparative role of SKP-SC-EVs was implicated in the activation of PI3K/Akt, mTOR, and p70S6k, as well as the reduction of Bax/Bcl-2 ratio, that was compromised by LY294002 to some extent. In addition, transferring miR-21-5p mimics into sensory neurons could partly protect them from OGD-induced impairment.

**Conclusions:**

Sum up, SKP-SC-EVs could improve neurite outgrowth of DRG sensory neurons in physiological and pathological condition. Moreover, the in vitro therapeutic potential of SKP-SC-EVs on the survival and restoration of OGD-injured sensory neurons was evidenced to be associated with miR-21-5p contained in the small EVs and miR-21-5p/PTEN/PI3K/Akt axis.

**Graphic abstract:**

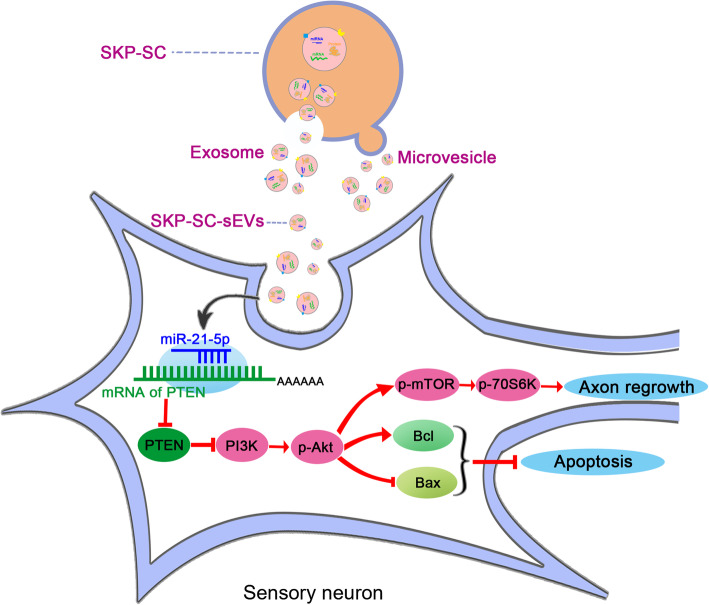

**Supplementary Information:**

The online version contains supplementary material available at 10.1186/s13287-020-02125-4.

## Background

In peripheral nervous system, Schwann cells (SCs) have been considered as supporting glial cells for neurons, facilitating the physiological process of neuro-regeneration spontaneously after mild nerve injury [1, 2]; however, the problems of poor regeneration may be attributed to the lack of abundant endogenous SCs and sufficient response, resulting in neurological dysfunction after severe nerve damage [3, 4].

Cell-based therapy is a promising strategy for nerve repair and protection in recent years, especially the application of Schwann-like cells differentiated from different sources of stem/progenitor cells, including adult stem cells [5]. For instance, mesenchymal stem/stromal cells (MSCs), resident in bone marrow, adipose, and umbilical cord, can be induced to differentiate into Schwann-like cells [6]. Notably, neural crest stem/precursor cells from postnatal bone marrow, dental pulp, and skin show desirable potential to generate Schwann-like cell [7]. The Schwann-like cells can respond to tissue injury, similar to endogenous SCs, modulating neuronal activity and regulating tissue microenvironment homeostasis [8, 9]. During the development process, in vivo neural crest cells give rise to SCs; therefore, focus on Schwann-like cells from reliable neural crest cell source for neuroprotection and neuro-regeneration is a significant work.

Skin precursors (SKPs) are originated from migrating neural crest cells, and it has been reported that human and rodent SKPs could generate Schwann-like cells, namely SKP-derived SCs (SKP-SCs) [10]. Although a series of studies have revealed the function of SKP-SC in vivo, alleviating pathological alterations caused by chronic spinal cord injury [11], peripheral nerve demyelinating lesions, and acute and delayed peripheral nerve injury (PNI) [12, 13], the paracrine mechanisms of SKP-SCs are poorly understood [14].

On the one hand, paracrine molecular mechanisms are responsible to the therapeutic potential of cell-derived secretome [15], including soluble trophic factors, cytokines, and extracellular vesicles (EVs), that suppose a new paradigm towards cell-free therapeutic mode in regenerative medicine [16]. On the other hand, paracrine communication mostly depends on EVs that contain a plethora of contents [17], trafficking proteins and lipids, as well as nucleic acids, including mRNA, microRNA (miR) [18, 19], and long non-coding RNA (lncRNA) [20, 21]. Furthermore, EVs have attracted the attention of the scientific community in recent years due to their widespread distribution and their great potential to be applied as therapeutic agents [22]. Therefore, development of cell-free therapies for peripheral nerve regeneration has been boosting [23, 24]. In particular, EVs from stem/progenitor cells and derivatives are providing the similar effect as cell therapy, while obviating the need of transplanting large number of cells and overcoming the poor survival of cell therapy, and guaranteeing the safety with effective targeting and faster clearance [25].

Accordingly, it was hypothesized that EVs derived from SKP-SCs (SKP-SC-EVs) would show significant potential equivalent to that of SKP-SCs, enhancing the restoration of damaged neural cells, which indicates promising application of SKP-SC-EVs on the neural injury result from mechanical, biochemical, and/or ischemic causative factors.

Previous study has shown that SKP-SCs could promote sensory recovery after cell therapy to PNI, resembling the effect of naive SCs [26]. Here, we utilized primarily cultured rat sensory neurons from dorsal root ganglions (DRGs) to simulate the ischemic and hypoxic lesions of damaged nerve [27]. The oxygen-glucose-deprivation (OGD) environment in vitro caused the alterations of neuronal morphology, cell viability, and expression of functional proteins. After SKP-SC-EV treatment, the neurite outgrowth and survival of neurons were investigated, and the phosphoinositide 3-kinase (PI3K) molecule signaling pathway was detected. Moreover, miR can bind to target gene mRNA and trigger the degeneration or post-translation inhibition resulting in protein dysfunction [28, 29]. Arising studies have reported that miR plays a vital role in the growth of development nerve and regrowth of damaged nerve [19]. According to reported experimental data, several miRs with potential of regulating PI3K pathway were screened, and their expression level was examined in sensory neurons after SKP-SC-EV treatment. Finally, miR-21-5p contained in SKP-SC-EVs was functionally verified.

## Methods

### Rat primary SKPs culture and differentiation into SCs

SKPs were isolated and cultured as previously described [30]. Briefly, back skin tissue was dissected from neonatal (1day) Sprague Dawley rats, and cut into 1 to 2 mm^2^ pieces followed by digestion with collagenase type XI (Sigma); the collected cells were plated at a density of 2–5 × 10^4^ cells/ml in proliferation medium, namely DMEM/F-12 (Corning) medium containing 40 ng/ml basic fibroblast growth factor (FGF, R&D), 20 ng/ml epidermal growth factor (EGF, R&D), 2% B27 (Gibco), and 1% antibiotics (penicillin/streptomycin, Gibco). When the sphere became large, SKPs were passaged with collagenase type XI digestion and sub-cultured in mixed medium (50% proliferation medium and 50% conditioned medium from SKPs).

To differentiate SKPs into SCs, spheres were digested into single cells, and inoculated on poly-d-lysine/laminin (Sigma) coated plates in SKP proliferation medium with 10% fetal bovine serum (FBS, Gibco) for 3 days. Then, the medium was changed to SKP differentiation medium, namely DMEM/F-12 (v/v: 3:1) medium containing 2% N2 (Stem Cell), 50 ng/ml heregulin-1β (R&D), 5 μM forskolin (Sigma), 1–3% FBS, and 1% antibiotics (penicillin/streptomycin), and the differentiation medium was changed every 3–4 days. SC colonies were separated into new petri dishes after 2 to 3 weeks using a cloning cylinder (Corning, USA) as previously described [30] (see Additional file 1: Supplementary Method). Moreover, SKP-SCs were cryopreserved at early passages for experimental application.

### Immunofluorescence staining

SKP spheres were fixed with 4% paraformaldehyde, blocked, and then incubated with primary antibodies, including mouse anti-Nestin (1:300, Abcam), rabbit anti-Versican (1:400, Sigma), and rabbit anti-Ki67 (1:400, Abcam) overnight at 4 °C, followed by reaction with FITC-anti-mouse-IgM (1:400, Proteintech), Cy3-anti-rabbit- IgM (1:400, Invitrogen), and Hoechst 33342 (Abcam) counterstaining. The cell samples were observed under a confocal laser scanning microscope (Zeiss, Germany).

SKP-SCs were subjected to immunofluorescence staining with rabbit anti-S100β (1:400, Invitrogen), rabbit-anti-p75 (1:500, CST), and chicken-anti-glial fibrillary acidic protein (anti-GFAP, 1:1000, Abcam), respectively, followed by the same reaction with the second antibody and Hoechst 33342 counterstaining.

For experimental application, the purified and cryopreserved SKP-SCs were recovered, then cells in each passage were subjected to immunofluorescence staining with S100β and Hoechst, and more than 300 cells were analyzed in each experiment (*n* = 3). The S100β positive cells were counted, and the percentage was calculated.

### EVs isolation, identification, and labeling

EVs were purified from the cell culture supernatant of SKP-SCs that were cultured in differentiation medium. After the cell density reached 80% confluence, medium was switched to serum-free medium for 48 h. The supernatant, namely conditioned medium, was collected and went through sequential ultracentrifugation at 500*g* for 10 min to remove the cell debris, followed by filtering through a 0.22-μm filter (Millipore). EVs were isolated using the exoEasy Kit (QIAGEN) from 15 ml cell culture supernatant; the detail experiment steps were following the manufacturer’s protocol.

The morphology of the EVs was observed under transmission electron microscope (TEM, Hitachi), and nanoparticle tracking analysis (NTA, German particle Metrix) was utilized to measure EV diameter and particle number. The protein concentration was measured using Bicinchoninic Acid (BCA) protein assay (Thermo Scientific), and exosomal markers CD9, CD63, and CD81 as well as tumor susceptibility gene 101 (TSG101) were detected by Western blot analysis.

Fluorescence labeling of EVs was performed according to the protocol previously described [24]. The PKH67 kit (Sigma) was used according to the instruction manual. The labeled EVs were concentrated by an Amicon Ultra 10 kDa tube (Millipore), and the EV uptake experiment was performed.

### DRG explant culture and sensory neuron culture with SKP-SC-EV treatment

DRGs were obtained from SD rat embryos (14.5 days). Briefly, DRGs were isolated and cultured on coverslips coated with poly-lysine in neurobasal medium (Gibco) with 2% B27, 1% mM l-glutamine, 50 ng/ml human nerve growth factor (NGF, R&D), and 1% penicillin-streptomycin. Besides, 10 mM cytosine arabinoside (Sigma) was added to remove non-neuronal cells. Then, low (1 × 10^8^ particles/ml), medium (2 × 10^8^ particles/ml), and high (4 × 10^8^ particles/ml) dose of EVs and PBS (control) were added to neurons daily during 5 days.

Moreover, DRG sensory neurons were primarily cultured as previously described [31]. Briefly, DRGs were collected from neonatal rats (1 day), then digested sequentially in 0.1% collagenase type I (Sigma) for 30 min and 0.25% trypsin (Gibco) for 20 min; then, single cells were collected and purified by centrifugation in PBS solution with 15% Bovine Serum Albumin (Sigma) before 10 mM cytosine arabinoside was added to remove non-neuronal cells. The obtained sensory neurons were cultured on the coated plates in medium the same as DRG explant culture medium, with treatment of medium-dose EVs (2 × 10^8^ particles/ml) for 12 h.

After the immunofluorescence staining of β-tubulin III (TUJ1, Abcam, 1:500), the axonal area of each DRG explant and the neurite length of single sensory neuron were measured with Image J software.

### OGD-injured sensory neurons and treatment of SKP-SC-EVs

OGD of sensory neurons was performed as previously described [9]. Briefly, sensory neurons were cultured in glucose-free neurobasal medium (Gibco) in 37 °C incubator with 5% CO_2_ and 1% oxygen concentration for 8 h, and switched back to their original culture condition with treatment of low-, medium-, and high-dose EVs for 24 h; additionally, in sequent experiments, high-dose EVs were applied and PI3K inhibitor LY294002 (CST) was added to medium. The morphology of DRG neurons was observed under phase-contrast microscope, and the neurite length and branch were analyzed by Image J software after immunofluorescence staining for TUJ1.

### Cell viability assay

The cell viability of sensory neurons was measured by cell counting kit-8 (CCK8, Dojindo) assay. The medium was mixed with CCK8 solution in a ratio of 10:1, then added to cells in different groups, and incubated at 37 °C for another 4 h. The value of optical density was measured at 450 nm using a microplate reader (BIOTEK). Cell viability is displayed as a percentage value relative to the control group.

### TUNEL staining

According to the TUNEL staining step of the kit for adherent cells (Promega), cells were fixed in 4% paraformaldehyde at 4 °C for 25 min, followed by permeabilization with 0.2% Triton X-100 for 10 min, then covered with equilibration buffer. Recombinant TdT was added to the equilibration buffer to avoid exposure to light, and the negative control was incubated with recombinant TdT-free culture buffer for 1 h. After termination of the reaction with 2 × SSC, nuclear staining was performed with DAPI at room temperature in the dark. Apoptotic (TUNEL positive) cells were detected as blue local bright green cells under a laser confocal microscope. To evaluate the cell apoptosis, we calculated the ratio of DAPI/TUNEL double-positive cells to DAPI-positive cells.

### Quantitative reverse transcriptase-polymerase chain reaction (qRT-PCR)

Total RNA was isolated from cells using TRIzol reagent (QIAGEN), and cDNA was obtained using Omniscript RT kit (QIAGEN) according to the manufacturer’s instructions. qRT-PCR was performed with SYBR Premix (QIAGEN) on the BIO-RAD system (BIO-RAD-96CFX) according to standard methods. Stem-loop RT primers (QIAGEN) were used to quantify the expression of miRNA, and the expression of U6 was used for standardization. The specific method was based on the manufacturer’s instruction, and miRNA primers were ordered from Ribobio. The results were analyzed by the 2^-△△Ct^ method.

### Cell transfection

The mimic of miR-21-5p and the negative control (NC) were transfected into sensory neurons with ribo-FECT transfection kit (Ribobio). The detailed experimental steps were following the manufacturer’s protocol.

### Western blot analysis

The protein samples from sensory neurons in different groups were extracted by using lysis buffer (Thermo Fisher Scientific). The concentration of protein samples was quantified, and equal amounts of protein were separated on 10–12% SDS-PAGE and transferred to PVDF membranes (Millipore), which were blocked with 5% non-fat milk in Tris-HCl buffered saline for 1 h at room temperature and incubated with primary antibodies, including CD9 (1:1000, Abcam), CD63 (1:1000, Abcam), CD81 (1:500, Sigma), TSG101 (1:500, Proteintech), β-actin (1:2000, Abcam), GAP43 (1:1000, Abcam), phosphate-protein kinase B (p-Akt) (1:1000, CST), total-Akt (t-Akt, 1:1000, CST), phosphate-mammalian target of rapamycin (p-mTOR, 1:1000, CST), total-mTOR (t-Mtor, 1:1000, CST), phosphate-P70S6K (p-P70S6K, 1:1000, CST), total-P70S6K (t-P70S6K, 1:1000, CST), Bax (1:1000, Abcam), Bcl-2 (1:1000, Proteintech), and glyceraldehyde-3-phosphate dehydrogenase (GAPDH, 1:1000, Abcam) at 4 °C overnight. The secondary antibody conjugated with horseradish peroxidase (1:5000, Abcam) was incubated with membranes for 2 h at room temperature.

### Statistical analysis

All quantitative data were presented as mean ± SEM from at least three independent experiments. And the statistical significance was analyzed using GraphPad Prism 8.0 software. Comparison between groups was assessed by one-way analysis of variance (ANOVA) and Tukey’s test, and *p* < 0.05 was considered statistically significant.

## Results

### Characterization of SKPs and SKP-SCs

**Fig. 1:**
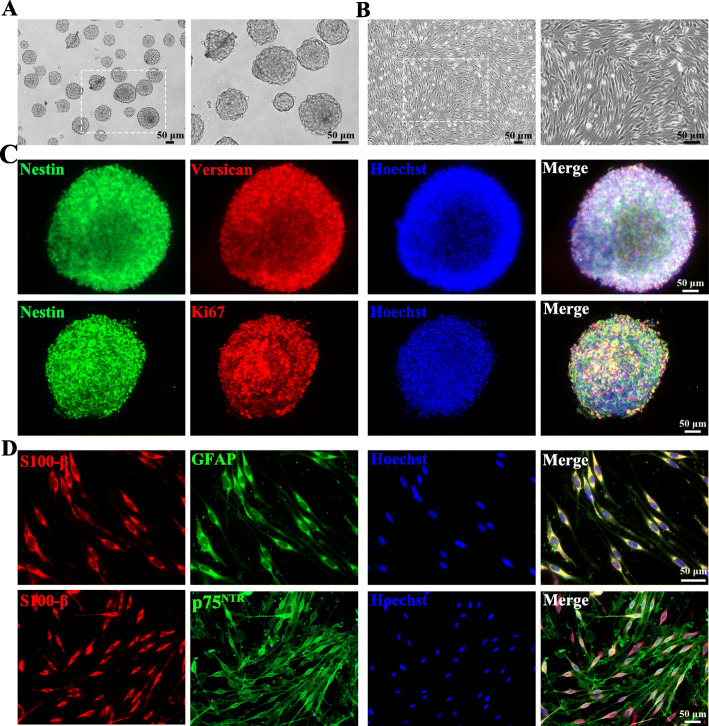
Characterization of SKPs and SKP-SCs. **a** SKPs in floating culture showed spherical colonies. **b** SKP-derived SCs showed spindle-like shape with side-by-side alignment. **c** Immunofluorescence staining of SKP spheres showed positive expression of intermediate filament Nestin (green) and chondroitin sulfate proteoglycan Versican (red) with proliferation marker Ki67 (red) and Hoechst (blue) labeled cell nuclei. **d** SKP-derived SCs showed positive expression of SC markers S100 (red), GFAP (green), and p75^NTR^ (green), and cell nuclei were labeled with Hoechst (blue). Scale bar, 50 μm

SKPs expanded in culture were growing as floating spherical colonies in vitro (Fig. 1a). Immunofluorescence analysis confirmed the positive expression of ectodermal neural stem cell marker Nestin and the hair follicle dermal papillary dermal sheath marker Versican, and the co-expression of proliferation marker Ki67 (Fig. 1c). It suggested that SKPs could sustain the proliferative capacity under in vitro culture condition.

Induction of differentiation of SKPs showed that the SKP-SCs displayed long-spindle morphology and side-by-side alignment (Fig. 1b), and positive expressed SC-specific markers, including S100β, GFAP, and p75^NTR^ (Fig. 1d). Results showed that the average purity of SKP-SCs was 98.5% (S100β positive percentage) in this work. Here, SKP-SCs in passage 15 to passage 17 were used for experiments.

### Isolation and identification of SKP-SC-EVs

**Fig. 2:**
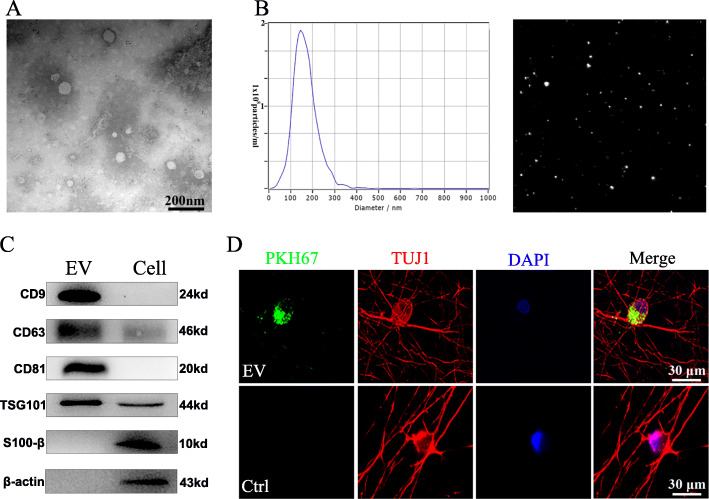
Characterization of SKP-SC-derived EVs. **a** Representative TEM image of SKP-SC-EVs presenting a typical cup-like concavity shape. Scale bar, 200 nm. **b** Representative traces from nanoparticle tracking analysis for SKP-SC-EVs. **c** Western blots showing the positive expression of exosomal markers CD9, CD63, CD81, and TSG101 in EVs, and β-actin and S100β were used as control markers in SKP-SCs. **d** PKH67 labeled EVs (green) were showing within the cytoplasm and axon of TUJ1 positive sensory neurons (red), and nuclei were labeled with DAPI (blue). Scale bar, 30 μm

Negative staining and TEM displayed that most EVs exhibited typical spherical bilayer membrane structure with cup-like concavity shape (Fig. 2a). The results from NTA demonstrated that the average diameter of these vesicles was 140.5 nm and the main peak of the particle size was located at 148.9 nm, and the concentration was about 1.6 ± 0.2 × 10^11^ particles/ml (Fig. 2b). Moreover, the EVs’ positive markers CD81, CD63, CD9, and Tsg101 were also expressed in SKP-SC-EVs, and β-actin and S100β were used as control markers positively expressed in SKP-SCs (Fig. 2c). In addition, after PKH67-labeled EVs culturing with sensory neurons for 4 h, PKH67-positive signals were observed in the cytoplasm and protrusion positions of neurons, which suggested that SKP-SC-EVs can be internalized by sensory neurons (Fig. 2d).

### Promotion of DRG axonal outgrowth by SKP-SC-EVs

**Fig. 3:**
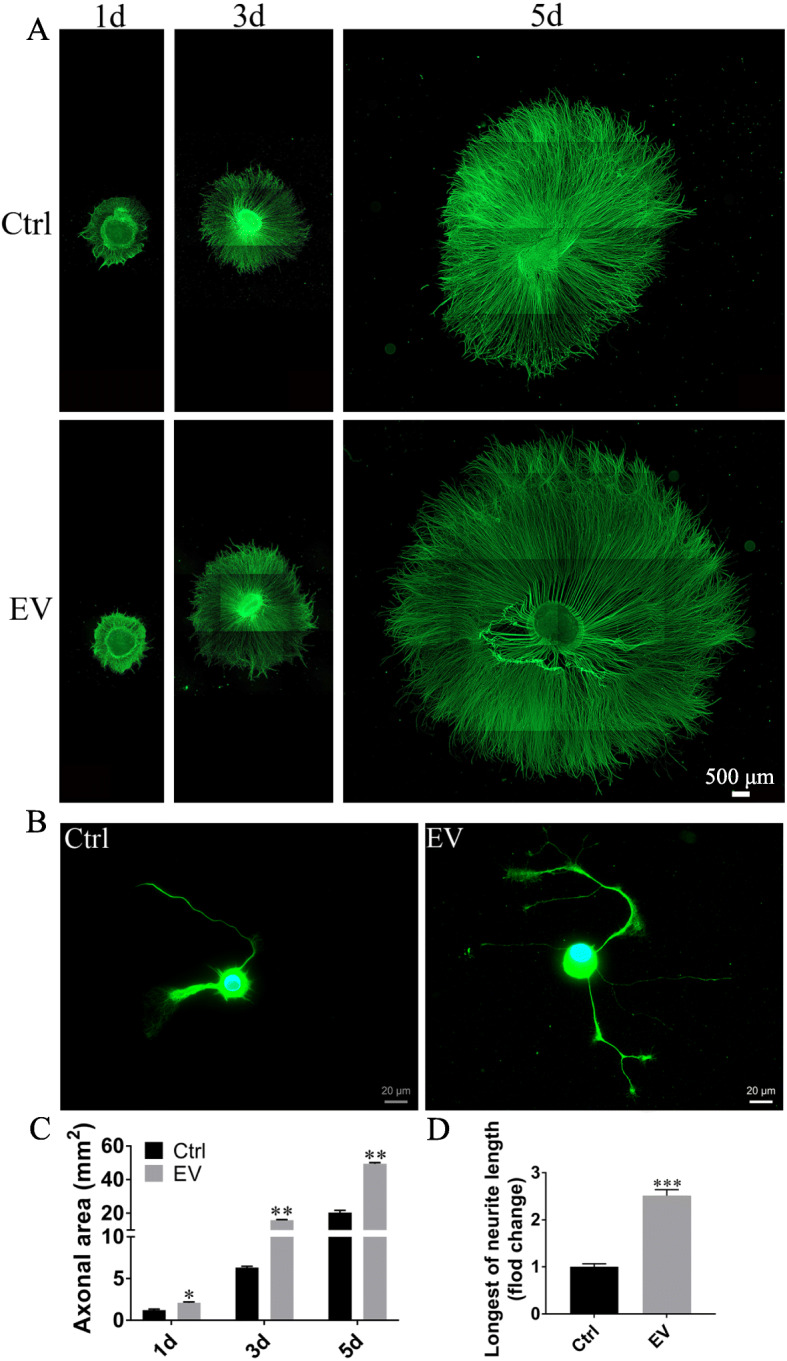
Effect of medium dose of SKP-SC-EVs on growth of DRGs and sensory neurons (see also Additional file 2: Figure S1). **a** Representative image of axonal outgrowth of TUJ1 positive (green) DRG explants after vehicle (Control) or EV treatment during 5 days. Scale bar, 500 μm. **b** TUJ1 (green) positive sensory neurons showing neurite growth after treatment with EVs for 12 h. Scale bar, 20 μm. **c** Histograms showing the increasing axonal area of DRGs in the EV groups. *n* = 5; *p < 0.05, **p < 0.01 compared with the vehicle group. **d** Histograms showing the longer neurite length of sensory neurons treated with EVs. ***p < 0.001 compared with the vehicle group

We evaluated whether SKP-SC-EVs could affect axonal outgrowth. Firstly, we examined the effect of SKP-SC-EVs on the axonal growth in DRG explant cultures. The results showed that, comparing with the control group, EV treatment at medium concentration for 1 day, 3 days, and 5 days significantly increased DRG explants’ axonal area (Fig. 3a, c). Moreover, we observed that three different concentrations of EVs all promoted DRG explants’ axonal growth comparing with the control group, while statistical analysis showed significant increase of neurite length in the middle- and high-dose treatment groups than in the low-dose treatment group (Additional file 2: Figure S1). The results suggested that DRGs do respond to EVs after the uptake of EVs into cells. Secondly, compared with the control group, EV treatment at medium concentration for 12 h significantly increased the neurite length of sensory neurons (Fig. 3b, d).

### Improvement of OGD-injured sensory neurons by SKP-SC-EVs via PI3K pathway

**Fig. 4:**
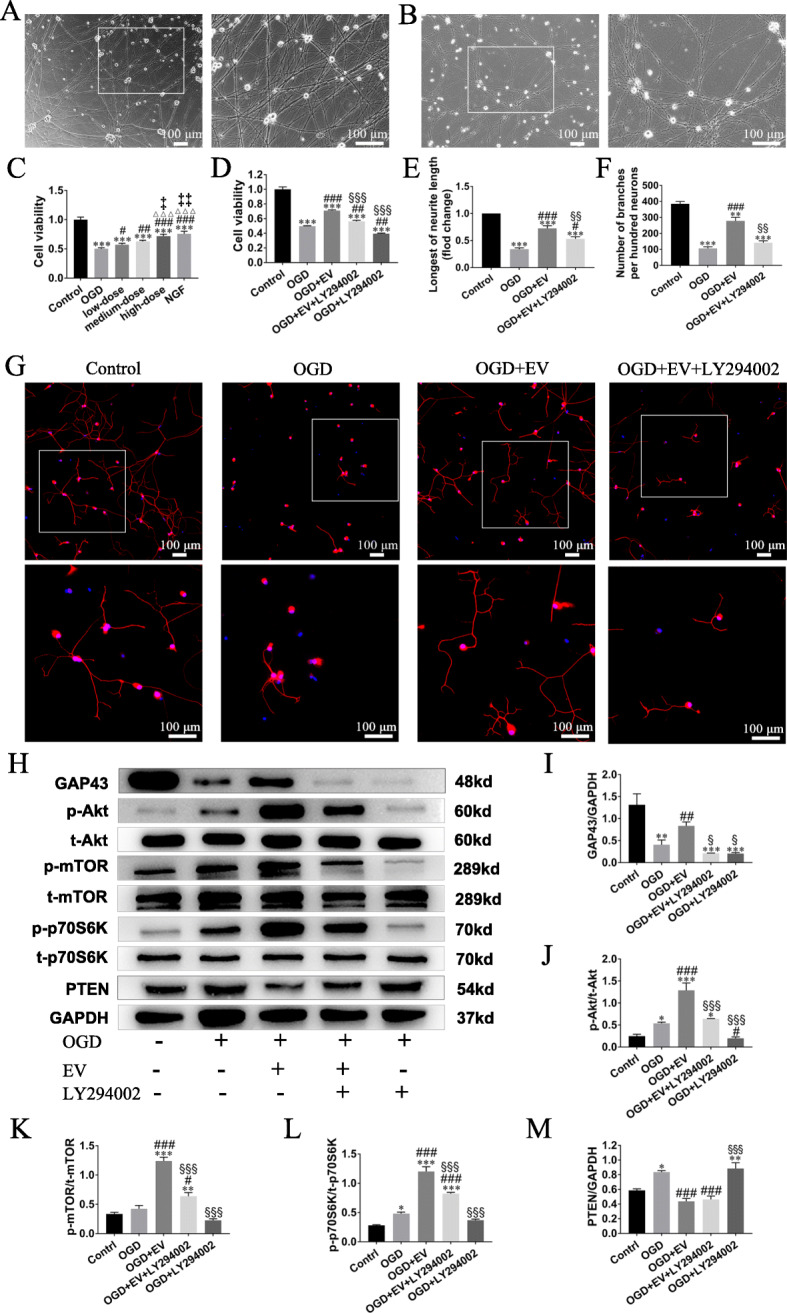
Treatment of OGD-injured sensory neurons resulted in neurite regrowth and pro-cell viability via molecule mechanism*.* **a** Neurites of sensory neurons extended and formed network 4 days after culture. Scale bar, 100 μm. **b** Neuron body shrinkage and neurite ruptures were shown in representative image 8 h after OGD. Scale bar, 100 μm. **c**, **d** Histograms showing the concentration-dependent effect of SKP-SC-EVs, and the inhibiting effect of LY294002 (PI3K blocker) on cell viability of OGD-injured neurons, and the neuronal viability in the high-dose EV group is equal to that in the NGF (positive control) group. **e**, **f** Statistical analysis of the longest neurite length and the number of branches per hundred cells. **g** Representative image showing neurite growth of sensory neurons in different groups with staining of TUJ1 (red), and nuclei were labeled with DAPI (blue). Scale bar, 100 μm. **h** Representative image of Western blot analysis for GAP43, p-Akt, p-mTOR, p-p70S6K, and PTEN expression in different groups. **i**–**m** Histograms showing increased expression level of GAP43, p-Akt, p-mTOR, and p-p70S6K in the OGD + EV group, and decreased expression level when LY294002 was added, while PTEN expression increased in the OGD group and decreased in the OGD + EV group. *n* = 3; ^△△△^*p* < 0.001, compared with the low-dose group; ^‡^*p* < 0.05, ^‡‡^*p* < 0.01, compared with the medium-dose group; *p < 0.05, **p < 0.01, ***p < 0.001 compared with the control group; ^#^*p* < 0.05, ^##^*p* < 0.01, ^###^*p* < 0.001 compared with the OGD group; ^§^*p* < 0.05, ^§§^*p* < 0.01, ^§§§^*p* < 0.001 compared with the OGD + EV group

Compared with DRG neurons cultured in regular medium (Fig. 4a), the injured neurons showed shrinking neuron soma and disrupted neurites at 8 h after exposure to OGD (Fig. 4b). At 24 h after treatment with different concentrations of EVs, sensory neurons showed markedly increased viability compared with OGD-injured neurons, and responded to EVs in a dose-dependent manner (Fig. 4c). Nevertheless, the increased viability of EV-treated sensory neurons can be inhibited by LY294002 (a blocker of PI3K pathway) (Fig. 4d).

Moreover, the immunofluorescence staining of TUJ1 for neurons was used to further analyze the growth of neurons (Fig. 4g). Compared with the OGD group, the average length of the longest neurites and the number of branches were significantly increased in the OGD + EV group, which was compromised by LY294002 to interrupt PI3K pathway. So that, neurons in the OGD + EV + LY294002 group exhibited shorter neurite length and fewer branches when compared with the OGD + EV group (Fig. 4e, f).

In addition, the expression level of GAP43 was significantly reduced compared with the control group; meanwhile, compared with the OGD group, the OGD + EV group reported higher expression level of GAP43; besides, after LY294002 was added, the expression level of GAP43 was reduced (Fig. 4h, i).

Furthermore, Western blot analysis showed the expression level of p-Akt, p-mTOR, p-P70S6K, and PTEN protein (Fig. 4h). Compared with the control group, their expression level increased in the OGD group; moreover, compared with the OGD group, their expression significantly increased after EV treatment, while after LY294002 added, their expression apparently decreased when compared with the OGD + EV group (Fig. 4j–l). PTEN expression increased in the OGD group and decreased in the OGD + EV group (Fig. 4m).

### Anti-apoptosis effect of SKP-SC-EVs on OGD-injured sensory neurons via regulating Bax/Bcl-2

**Fig. 5:**
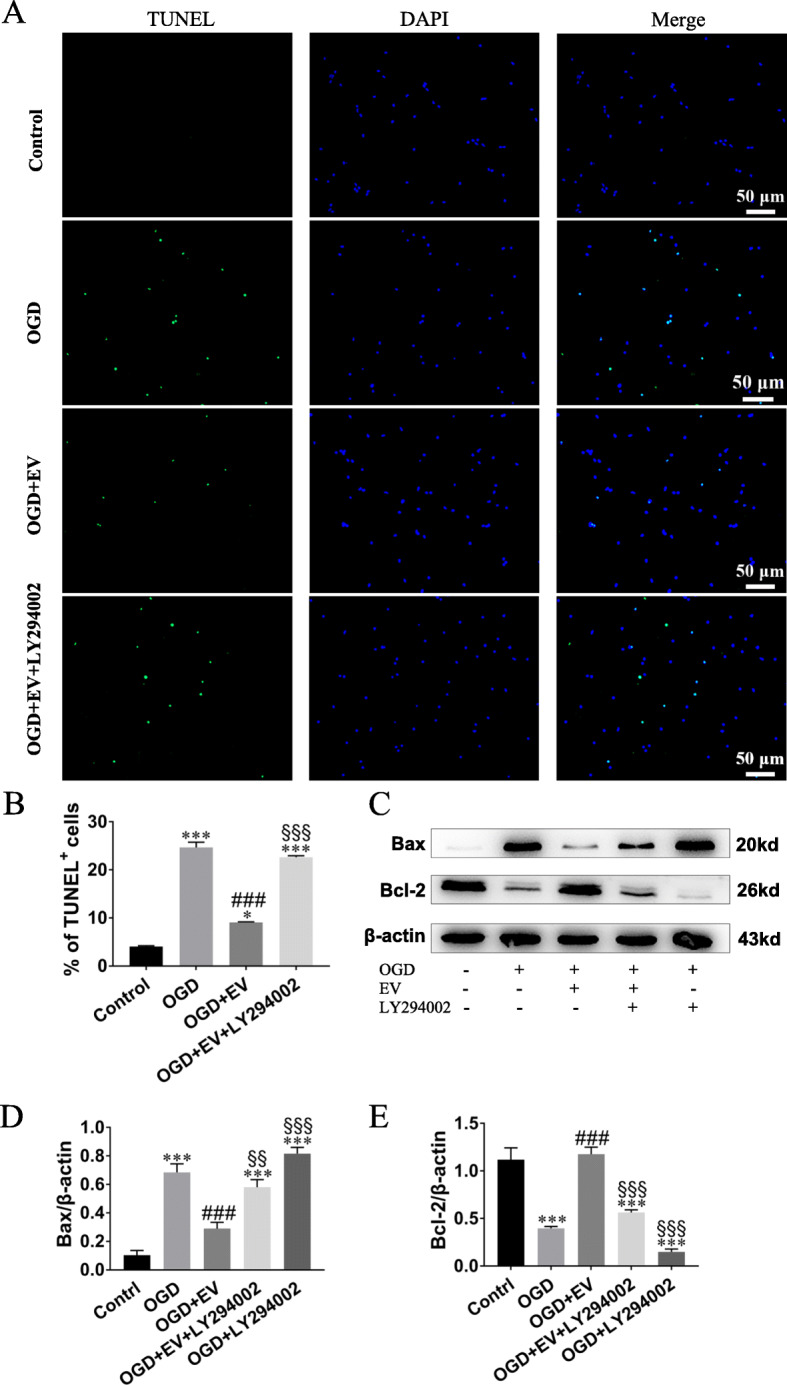
SKP-SC-EVs attenuated neuronal apoptosis after OGD. **a** Representative image of TUNEL staining of sensory neuron in different groups showing TUNEL positive (green) neurons in the control, OGD, OGD + EV, and OGD + EV + LY294002 groups, and DAPI (blue) labeled cell nuclei. Scale bar, 50 μm. **b** Histograms showing the percentage of TUNEL positive neurons in different groups. **c** Representative image of Western blot analysis for Bax and Bcl-2 expression in different groups. **d**, **e** Histograms showing decreased Bax expression level and increased Bcl-2 expression level after SKP-SC-EV treatment to OGD-injured sensory neurons, while the expression level of Bax and Bcl-2 was reversed by adding LY294002. *n* = 3; **p < 0.01, ***p < 0.001 compared with the control group; ^##^*p* < 0.01, ^###^*p* < 0.001, compared with the OGD group; ^§^*p* < 0.05, ^§§^*p* < 0.01, ^§§§^*p* < 0.001 compared with the OGD + EV group

After the exposure to OGD, TUNEL staining showed the deterioration of apoptosis of sensory neurons compared with the control group (Fig. 5a). Moreover, compared with the OGD group, EV treatment significantly ameliorated the apoptosis, although that was partially abolished by LY294002 (Fig. 5b).

In addition, Western blot showed that the expression level of Bax was higher in neurons exposed to OGD compared with the control group, but the expression level of Bcl-2 was lower (Fig. 5c); when compared with the OGD group, the expression level of Bax/Bcl-2 ratio in the OGD + EV group was significantly reversed; furthermore, when compared with the OGD + EV group, the effect of EVs was partly suppressed by LY294002 (Fig. 5d, e). The data above indicated that SKP-SC-EVs could mitigate neuron apoptosis via regulation of Bax/Bcl-2 expression ratio.

### Evidence of miR-21-5p contained in SKP-SC-EVs and potential action on OGD-injured sensory neurons

**Fig. 6:**
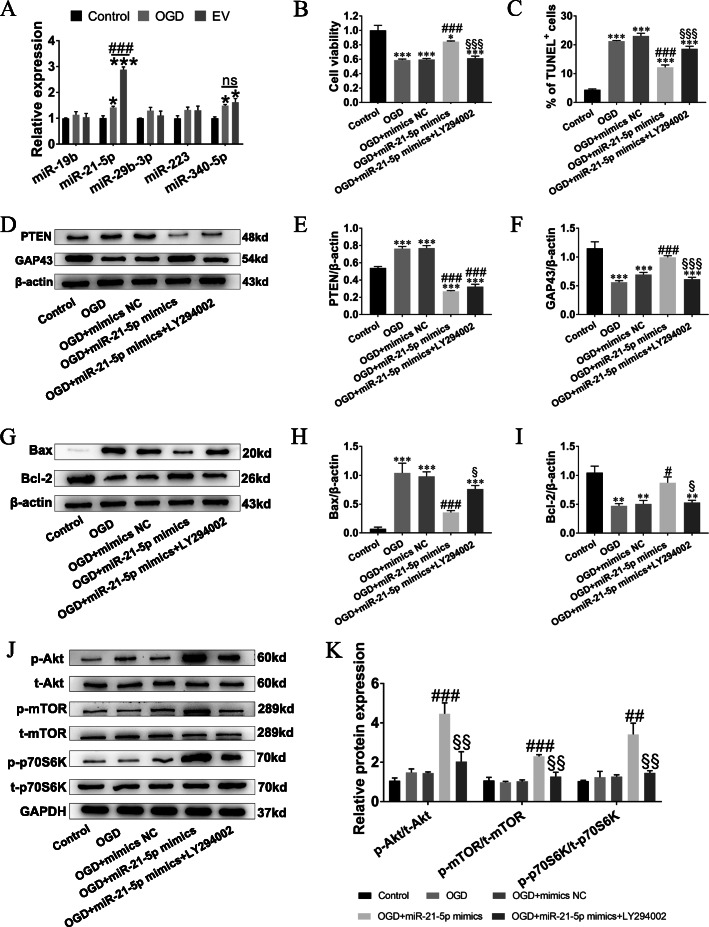
SKP-SC-EVs promoted neuronal viability and attenuated neuronal apoptosis via miR-21-5p negatively regulating PTEN-PI3K pathway. **a** Expression of miR-19b, miR-21-5p, miR-29b-3p, miR-223, and miR-340-5p, showing increased miR-21-5p in sensory neurons treated with SKP-SC-EVs after OGD. **b**, **c** Histograms showing the cell viability and the percentage of TUNEL positive neurons in different groups (see also Additional file 4: Figure S2). **d** Representative image of Western blot analysis for PTEN and GAP43 expression in different groups. **e**, **f** Histograms showing increasing expression level of PTEN and decreased expression level of GAP43 after treatment of OGD, while miR-21-5p overexpression reversed their expression, whereas GAP43 expression was abolished by LY294002. **g** Representative image of Western blot analysis for Bax and Bcl-2 in different groups. **h**, **i** Histograms showing decreased Bax expression level and increased Bcl-2 expression level after overexpression miR-21-5p in OGD-injured sensory neurons, while the expression level of Bax and Bcl-2 was reversed by adding LY294002. **j** Representative image of Western blot analysis for p-Akt, p-mTOR, and p-p70S6K expression in different groups. **k** Histograms showing increased expression level of p-Akt, p-mTOR, and p-p70S6K in the OGD + miR-21-5p mimics group, and decreased expression level when LY294002 was added. *n* = 3; *p < 0.05, **p < 0.01, ***p < 0.001 compared with the control group; ^#^*p* < 0.05, ^###^*p* < 0.001 compared with the OGD group; ^§^*p* < 0.05, ^§§^*p* < 0.01, ^§§§^*p* < 0.001 compared with the OGD + miR-21-5p mimics group

EVs are well documented to be enriched with miRs and can deliver miRs to recipient cells to regulate cellular functions. We examined several miRs related to neural repair and phosphatase and tensin homolog (PTEN)-PI3K pathway regulation, including miR-19b, miR-21-5p, miR-29b-3p, miR-223, and miR-340-5p. Our PCR results showed that after OGD, the expression of miR-21-5p and miR-340-5p was triggered in sensory neurons, but only the expression of miR-21-5p was significantly upregulated after treatment with SKP-SC-EVs, while the expression of miR-19b, miR-29b-3p, and miR-223 was almost unchanged (Fig. 6a). And qRT-PCR was employed to examine the quantity of microRNAs in EV, U6 was set as internal reference, and the CT value and △CT value were shown in Additional file 3: Table S1.

Next, we verified the potential action by transfection of miR-21-5p mimics into sensory neurons. After effective transfection of miR-21-5p was detected (Additional file 4: Figure S2), the pro-viability and anti-apoptosis effect of miR-21-5p were determined, results also showed that the promoting effect of miR-21-5p could be apparently abolished by LY294002 in OGD-injured neurons (Fig. 6b, c), and the representative images of TUNEL staining of different groups were shown (Additional file 5: Figure S3). Simultaneously, OGD treatment remarkably promoted PTEN expression level and inhibited GAP43 expression level; meanwhile, the alteration was significantly alleviated when miR-21-5p overexpressed; PTEN was not significantly attenuated by LY294002 in OGD-injured neurons, while GAP43 was significantly attenuated by LY294002 in OGD-injured neurons (Fig. 6d–f). These data indicated that SKP-SC-EVs could resume OGD-injured neurons through miR-21-5p negatively regulating PTEN.

### Regulation of downstream signaling molecules of PTEN-PI3K pathway via miR-21-5p contained in SKP-SC-EVs

In order to clarify the action mechanism of miR-21-5p protecting sensory neurons against OGD-induced cell injury, Western blot detected the expression level changes of apoptosis-related Bax and Bcl-2 (Fig. 6g). Results showed that the overexpression of miR-21-5p decreased the expression level of Bax and increased the expression level of Bcl-2 compared with the OGD group (Fig. 6h, i). As expected, the expression level of p-Akt, p-mTOR, and p-p70S6K protein increased, also could be abolished by LY294002 in OGD-injured neurons (Fig. 6j, k), which suggested that the overexpression of miR-21-5p imitated the activation of PI3K signaling pathway by SKP-SC-EV treatment.

## Discussion

**Fig. 7:**
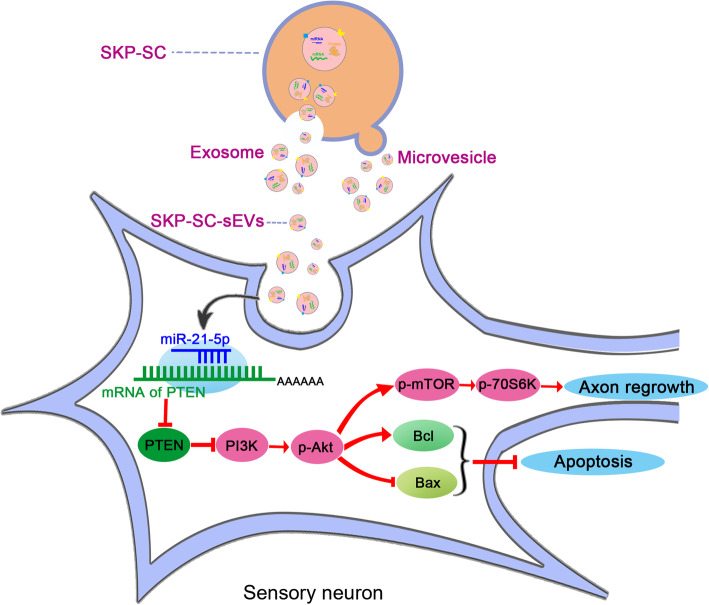
Graphic abstract: SKP-SC-EVs’ internalization and miR-21-5p/PTEN/PI3K/Akt axis in sensory neurons

Results from this study indicated that the internalization of SKP-SC-EVs could potentiate not only the neurite extension of regularly cultured sensory neurons, but also the neurite regrowth and cell survival of OGD-injured sensory neurons. When damages applied to neurons, the cells would require their own homeostasis regulation in the damage and anti-damage process. And whether the pathological alteration would occur was depending on the contrast between the damage force and anti-damage force. Here, OGD induced PTEN expression that is the unfavorable alteration with increase of TUNEL cells; simultaneously, OGD also evoked anti-damage compensatory regulating mechanism with increasing activation of Akt pathway. In the present work, the supplemented EVs can enhance the endogenous anti-damage force in sensory neurons through miR-21-5p targeting inhibiting PTEN that can increase the expression of signaling molecules in PI3K/Akt pathway, which primarily exerted the role of the classic pro-growth and pro-viability signaling pathway. We confirmed that the efficacy of SKP-SC-EVs was partially due to PI3K/Akt pathway activation, which improve neurite growth whereas attenuate apoptosis. It was further proved that SKP-SC-EVs can transfer miR-21-5p into sensory neurons, and miR-21-5p was able to target and inhibit PTEN gene, thereby regulating downstream signaling molecules in PI3K/Akt pathway, including mTOR and p70S6k activation, as well as the reduction of Bax/Bcl-2 ratio (see graphic abstract: Fig. 7).

To ensure the relative reproducibility of EVs’ functional assay for the exploration of their cell-free therapeutic potential, in this work, the exoEasy Maxi Kit with a membrane-based affinity binding column was used to purify exosomes and other EVs from culture supernatants of stable passaged of SKP-SCs. In particular, a generic, biochemical feature of vesicles was utilized to recover the entire spectrum of EVs present in the preparations [32]. Classically, in the group of heterogeneous particles, EVs were divided into three major classes, exosomes (30–150 nm in diameter), microvesicles (50–1000 nm in diameter), and apoptotic bodies (a less studied subgroup). Also, members of four academic societies have defined small EVs (50–200 nm in diameter) derived from MSCs for therapeutic applications [33, 34]. Furthermore, small EVs can be secreted from most, if not all, other types of cells [35, 36]. Accordingly, EVs from SKP-SCs (around of 148 nm diameter) are most of small EVs, including exosomes, displaying cup-like concavity under TEM, and expressing exosomal markers (CD9, CD63, CD81, and TSG101).

Recently, EVs have been characterized as vital mediators of paracrine communication, especially small EVs which can influence both physiological and pathological conditions [22, 33], which was also shown by SKP-SC-EVs in this study. In normal situation, the growth of DRGs and sensory neurons was enhanced by SKP-SC-EVs; additionally, after SKP-SC-EV treatment, OGD-injured neurons recovered the expression of functional protein GAP-43, neurite outgrowth, and cell viability. In agreement with this, EVs from SCs or other Schwann-like cells have been evidenced as messenger for cell-to-cell communication, involved in neurite growth and regeneration process. SC-derived exosomes can mediate neuron-glia communication and enhance axonal regeneration through inducing the neuronal growth cone formation [24, 37, 38]; moreover, Wang et al. provided evidence that SC-exosomes have a therapeutic effect on diabetic peripheral neuropathy in mice and suggested that SC-exosomes’ modulation of miRs contributes to this therapy [39]; especially, SCs reprogramming into repair cells can increase miR-21 expression in exosomes, promoting axonal growth [40]. In the same way, SC-like differentiated adipose stem cells could promote neurite outgrowth via secreted exosomes and RNA transfer [41].

In present work, we demonstrated that PTEN was upregulated in OGD-treated sensory neurons, and SKP-SC-EVs could inhibit neuronal apoptosis via block PTEN expression, then activate PI3K/Akt pathway; subsequently, this classic pathway further induces several downstream signaling molecules, involving in growth and death biological process homeostasis, which also could be suppressed by PI3K pathway blocker. Given as drug delivery vehicles, EVs may transfer a diversity of cargoes, including RNA, protein, and lipid; in particular, miRs show activity to regulate target genes and downstream pathways, leading to the alterations of cellular biological behavior. As it was reported previously, PTEN gene is one of the main negative regulators of PI3K/Akt pathway, and inhibition of PTEN can facilitate intrinsic regenerative outgrowth of adult peripheral axons [42]; from well-documented papers, we screened out several miRs that implicated with PTEN downregulation and neuronal repair. The literature on EVs suggested that miR candidates, such as miR-19b [43], miR-21 [40], miR-29b-3p [44], miR-233 [45], and miR-304-5p [46], would enable the activation of PI3K and downstream signaling molecules.

Then, high expression level of miR-21-5p was successfully detected out from sensory neurons after internalization of miR-enriched SKP-SC-EVs. Furthermore, miR-21-5p mimics inhibited PTEN expression and strengthened the repair effect on OGD-injured sensory neurons; the efficacy could also be abolished by PI3K inhibitor (LY294002). Consistently, recent investigations have reported the effect of miR-21 on alleviating optic nerve injury, spinal cord injury, ischemic brain injury, early brain injury, traumatic brain injury, and status epilepticus [47–52]. Besides to PI3K pathway that is classical to growth and apoptosis regulation, miR-21 can also modulate Wnt/beta-catenin signaling pathways [47] and FasL gene axis [52], while inactivate PDCD4/p21 pathway [53]. Moreover, miR-21-5p might regulate autophagy [51, 54], oxidative stress [53, 55], and inflammation [52, 56] to mediate neuroprotective and neuro-reparative effect.

In addition, other selected miRs showed unfavorable expression level in OGD-injured sensory neurons after SKP-SC-EV treatment, which indicate that the physicochemical properties and molecular-compound delivery of EVs are determined by their cell, tissue, or fluid of origin, and the heterogeneity present in the source is reflected in the composition of the EVs. Therefore, the properties can be harnessed by bioengineering, to improve their bioactivity, stability, targeting, and presentation of native EVs [57]. Furthermore, the testing of more candidate miRs derived from SKP-SCs is ongoing via high-throughput analysis, and should yield more useful information for the development of innovative cell-free therapy for nerve regenerative translational medicine from bench to bedside. Acting as potential alternatives to cell therapy, the advantages of EVs include convenient storage, stable function, and minor or absent immune-rejection [57]; especially, nanometer grade EVs have the ability to transfer bioactive components and surmount biological barriers (e.g., blood-brain barrier), so that they can be explored as potential therapeutic agents [33].

However, elucidating the complicated regeneration mechanisms is still in challenge, and there exist cross-talks involving distinct, complex multicellular responses that can guide and sustain axonal regrowth in peripheral nerve regeneration process [58]. For instance, Simeoli et al. have reported that exosomal cargo including miR could regulate sensory neuron to macrophage communication after nerve trauma [56], and MSC-derived exosomes could induce proliferation and migration of normal and chronic wound fibroblasts, and enhance angiogenesis [59]; moreover, the overexpression of miR-21-5p can prevent the oxidative stress-induced apoptosis of SCs themselves by suppressing autophagy [55].

Nonetheless, the regulation effect of EVs on PI3K and more pathways should not be confined in shutting miRs, because several defined lncRNAs have been reported to exert neuroprotective potential [60–62], and proteins enriched in SC-derived EVs also could act as bioactive factors once delivered to recipient cells, enhancing proliferation and migration as well as angiogenesis, while negatively regulating apoptosis and oxidative stress [63]. Therefore, more potential of SKP-SC-EVs such as axon guidance, pro-angiogenesis, promoting myelination, and immune inflammation modulation for neuro-regeneration microenvironment construction should be further investigated, depending on the genetic and proteomic contents. In addition, the limitation of the in vitro approach used to elucidate mechanisms must ultimately be addressed in vivo, and the therapy efficacy of the application of SKP-SC-EVs for the amelioration of nerve dysfunction also required to be verified in animal experiments.

## Conclusions

Taken together, these findings demonstrated the pro-growth activity of SKP-SC-EVs in both physiological and pathological conditions, as concerned to the pro-regrowth, pro-viability, and anti-apoptotic ability of SKP-SC-EVs that can be mediated through PI3K pathway in vitro*.* Moreover, miR-21-5p contained in SKP-SC-EVs played a significant role in the improvement of OGD-injured DRG sensory neurons through PTEN-PI3K pathway. In future work, we will focus on excavating more information to clarify the potential and action mechanism of SKP-SC-EVs on neuro-regeneration.

## Supplementary Information

**Additional file 1: Supplementary Method.** (Isolation/purification of SCs from SKPs).

**Additional file 2: Figure S1.** Effect of SKP-SC-EVs on DRG axonal outgrowth. **(A)** Representative images of DRG explants in different EVs concentration groups. Scale bar, 500 μm. **(B)** Histograms showing the difference of axonal area among low-, medium-, and high-dose EVs groups and control group (see also Fig. 3a). *n* = 4; *p < 0.05, compared with control group; ^###^*p* < 0.001 compared with low-dose EVs group.

**Additional file 3: Table S1.** CT value and △CT value of microRNAs in SKP-SC-EVs.

**Additional file 4: Figure S2.** Transfection efficiency of miR-21-5p mimics into sensory neurons. The histogram showing that the relative miR-21-5p expression in mimics transfection group is significantly higher than that in the control group and the mimic NC group. *n* = 3; *** *p* < 0.001 compared with control group.

**Additional file 5: Figure S3.** TUNEL staining of cultured sensory neurons after treatment of miR-21-5p. Representative images showing TUNEL positive (green) neurons in control, OGD, OGD+mimics NC, OGD+miR-21-5p mimics and OGD+miR-21-5p mimics+LY294002 groups, and DAPI (blue) labeled cell nuclei (see also Fig. 6c). Scale bar, 50 μm.

## Data Availability

All of the data and materials are available upon reasonable request.

## Abbreviations

Akt: Protein kinase B
CCK8: Cell counting kit-8
DRG: Dorsal root ganglion
EGF: Epidermal growth factor
EV: Extracellular vesicle
FBS: Fetal bovine serum
FGF: Basic fibroblast growth factor
GAPDH: Glyceraldehyde-3-phosphate dehydrogenase
miR: MicroRNA
mTOR: Mammalian target of rapamycin
NGF: Nerve growth factor
NTA: Nanoparticle tracking analysis
OGD: Oxygen-glucose-deprivation
PI3K: Phosphoinositide 3-kinase
PNI: Peripheral nerve injury
PTEN: Phosphatase and tensin homolog
SC: Schwann cell
SKP: Skin precursor
TEM: Transmission electron microscopy
TSG101: Tumor susceptibility gene 101
TUJ1: β-Tubulin III
