# Supplementary material for: Improvement of sensory neuron growth and survival via negatively regulating PTEN by miR-21-5p-contained small extracellular vesicles from skin precursor-derived Schwann cells
Source: Stem Cell Res Ther. 2021 Jan 25;12:80. doi: 10.1186/s13287-020-02125-4 (PMC7831194; doi:10.1186/s13287-020-02125-4)
Supplement: Supplementary file 1 — Additional file 1: Supplementary Method. (Isolation/purification of SCs from SKPs). [file 13287_2020_2125_MOESM1_ESM.pdf]

Supplementary Methods

Isolation/purification of SCs from SKPs

SC colonies were separated into new petri dishes after 2 to 3 weeks. To generate large numbers of purified SKP-derived Schwann cells, we isolated SC colonies mechanically using a cloning cylinder(Corning, USA). Briefly, we sterilize cloning cylinders by autoclaving, and place a pen mark on the underside of the dish to identify the locations of these colonies under the tissue culture microscope, then remove the medium from the dish, place the cylinder over an individual SC colony. Fill the cylinder with 200–400μl of trypsin EDTA and incubate at 37°C for 2–3 min, cells should then be easily detached from the dish with pipette. Remove all medium from the cylinder into culture tube and dilute the cell suspension in 10 ml of wash medium containing 10% FBS to inactivate the enzyme and then pellet cells by centrifugation in a tabletop centrifuge at 1,100 rpm. for 5–7 min. Cells can then be resuspended in SKPs differentiation medium. Plate the resuspended cells in laminin/poly-D-lysine-coated dishes, and place them in a 37 ℃, 5% CO_2 incubator, fed fresh differentiation medium every 2–3 d. When they reach confluence, passage SKP derived SCs.
