# Supplementary material for: Improvement of sensory neuron growth and survival via negatively regulating PTEN by miR-21-5p-contained small extracellular vesicles from skin precursor-derived Schwann cells
Source: Stem Cell Res Ther. 2021 Jan 25;12:80. doi: 10.1186/s13287-020-02125-4 (PMC7831194; doi:10.1186/s13287-020-02125-4)
Supplement: Supplementary file 2 — Additional file 2: Figure S1. Effect of SKP-SC-EVs on DRG axonal outgrowth. (A) Representative images of DRG explants in different EVs concentration groups. Scale bar, 500 μm. (B) Histograms showing the difference of axonal area among low-, medium-, and high-dose EVs groups and control group (see also Fig. 3a). n = 4; *p < 0.05, compared with control group; ###p < 0.001 compared with low-dose EVs group. [file 13287_2020_2125_MOESM2_ESM.pdf]

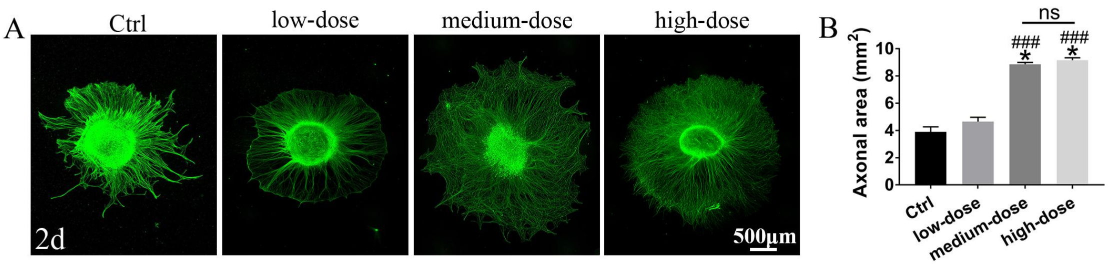

Fig. S1 Effect of SKP-SC-EVs on DRG axonal outgrowth. **(A)** Representative images of DRG explants in different EVs concentration groups. Scale bar, 500 µm. **(B)** Histograms showing the difference of axonal area among low-, medium-, and high-dose EVs groups and control group. n = 4; *$p < 0.05$, compared with control group; ###$p < 0.001$ compared with low-dose EVs group.
