# Supplementary material for: Improvement of sensory neuron growth and survival via negatively regulating PTEN by miR-21-5p-contained small extracellular vesicles from skin precursor-derived Schwann cells
Source: Stem Cell Res Ther. 2021 Jan 25;12:80. doi: 10.1186/s13287-020-02125-4 (PMC7831194; doi:10.1186/s13287-020-02125-4)
Supplement: Supplementary file 3 — Additional file 3: Table S1. CT value and △CT value of microRNAs in SKP-SC-EVs. [file 13287_2020_2125_MOESM3_ESM.pdf]

**Table S1** CT value and △CT value of microRNAs in SKP-SC-EVs

| microRNA | CT value (means ± SEM) | △ CT value (means ± SEM) |
|---|---|---|
| miRNA-19b | 33.02 ± 0.87 | 15.86 ± 0.98 |
| miRNA-21-5p | 20.24 ± 0.36 | 3.08 ± 0.48 |
| miRNA-29b-3p | 31.54 ± 0.40 | 14.08 ± 0.51 |
| miRNA-223 | 30.07 ± 0.41 | 12.91 ± 0.30 |
| miRNA-340-5p | 30.49 ± 0.45 | 13.33 ± 0.34 |
| U6 | 17.16 ± 0.11 | 0 |
