# Supplementary material for: Improvement of sensory neuron growth and survival via negatively regulating PTEN by miR-21-5p-contained small extracellular vesicles from skin precursor-derived Schwann cells
Source: Stem Cell Res Ther. 2021 Jan 25;12:80. doi: 10.1186/s13287-020-02125-4 (PMC7831194; doi:10.1186/s13287-020-02125-4)
Supplement: Supplementary file 4 — Additional file 4: Figure S2. Transfection efficiency of miR-21-5p mimics into sensory neurons. The histogram showing that the relative miR-21-5p expression in mimics transfection group is significantly higher than that in the control group and the mimic NC group. n = 3; *** p < 0.001 compared with control group. [file 13287_2020_2125_MOESM4_ESM.pdf]

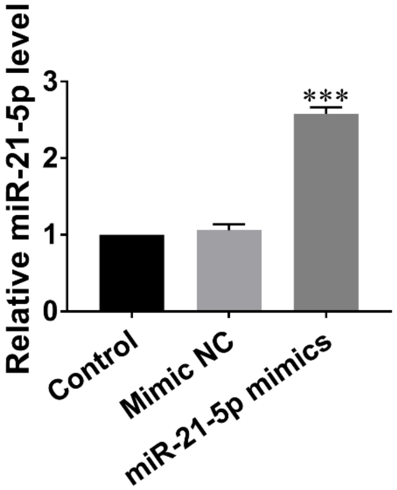

Fig. S2 Transfection efficiency of miR-21-5p into sensory neurons. The histogram showing that the relative miR-21-5p expression in mimics transfection group is significantly higher than that in the control group and the mimic NC group. $n = 3$; *** $p < 0.001$ compared with control group.
