# Supplementary material for: Improvement of sensory neuron growth and survival via negatively regulating PTEN by miR-21-5p-contained small extracellular vesicles from skin precursor-derived Schwann cells
Source: Stem Cell Res Ther. 2021 Jan 25;12:80. doi: 10.1186/s13287-020-02125-4 (PMC7831194; doi:10.1186/s13287-020-02125-4)
Supplement: Supplementary file 5 — Additional file 5: Figure S3. TUNEL staining of cultured sensory neurons after treatment of miR-21-5p. Representative images showing TUNEL positive (green) neurons in control, OGD, OGD+mimics NC, OGD+miR-21-5p mimics and OGD+miR-21-5p mimics+LY294002 groups, and DAPI (blue) labeled cell nuclei (see also Fig. 6c). Scale bar, 50 μm. [file 13287_2020_2125_MOESM5_ESM.pdf]

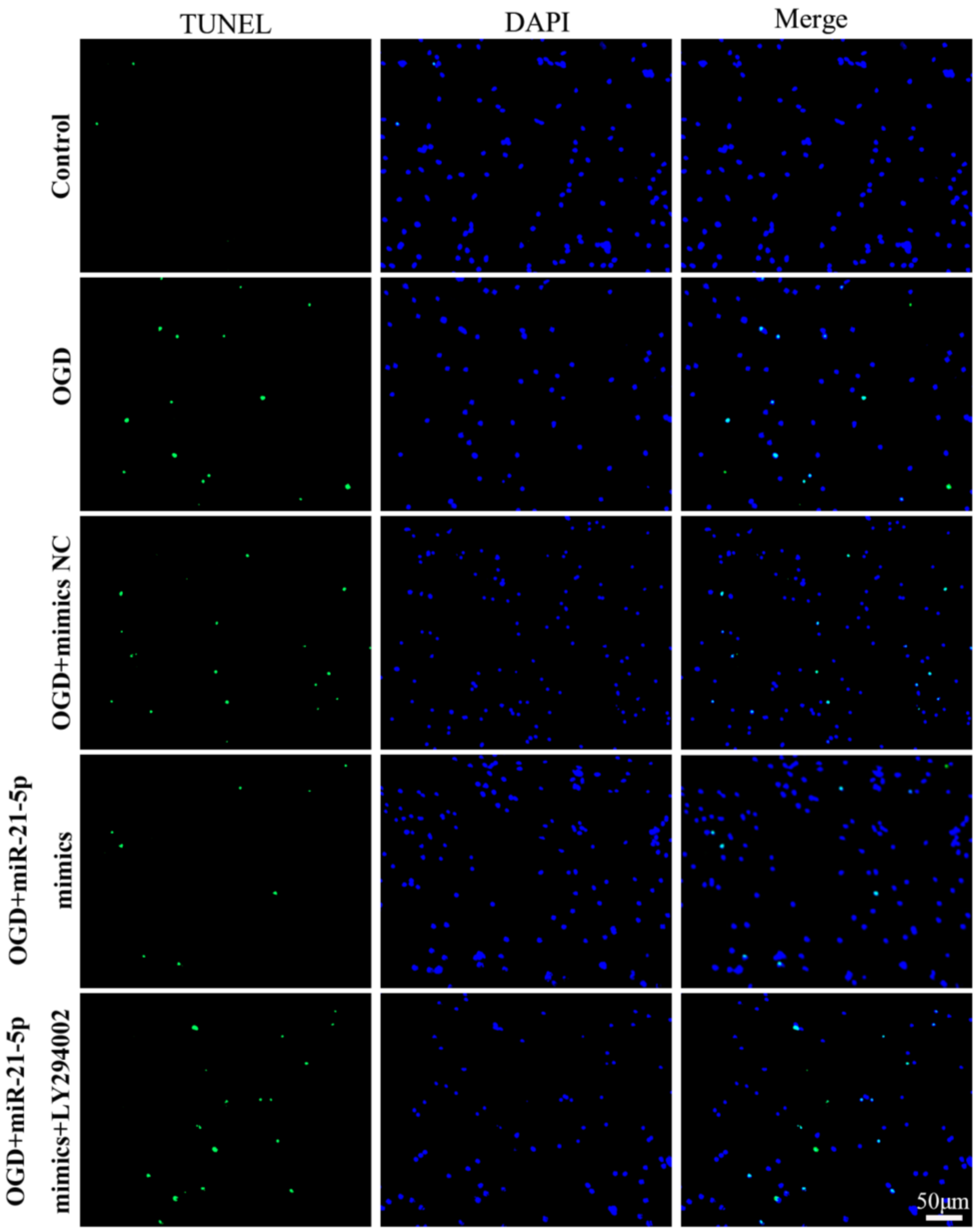

Fig. S3 TUNEL staining of cultured sensory neurons after treatment of miR-21-5p. Representative images showing TUNEL positive (green) neurons in control, OGD, OGD+mimics NC, OGD+miR-21-5p mimics and OGD+miR-21-5p mimics+LY294002 groups, and DAPI (blue) labeled cell nuclei (see also Fig. 6C). Scale bar, 50 μm.
